# Optimization of Ultrasound-Assisted Extraction of Natural Antioxidants from the *Osmanthus fragrans* Flower

**DOI:** 10.3390/molecules21020218

**Published:** 2016-02-18

**Authors:** An-Na Li, Sha Li, Ya Li, Dong-Ping Xu, Hua-Bin Li

**Affiliations:** 1 Guangdong Provincial Key Laboratory of Food, Nutrition and Health, School of Public Health, Sun Yat-Sen University, Guangzhou 510080, China; lianna28@163.com (A.-N.L.); saferide@126.com (Y.L.); xudongping1989@163.com (D.-P.X.); 2 School of Chinese Medicine, The University of Hong Kong, Hong Kong, China; lishasl0308@163.com

**Keywords:** *Osmanthus fragrans*, flower, antioxidant, ultrasound-assisted extraction, response surface methodology

## Abstract

An ultrasound-assisted extraction (UAE) method was developed to extract natural antioxidants from the *Osmanthus fragrans* flower. The effect of UAE on antioxidant activity of the extract from the *Osmanthus fragrans* flower was studied using a Trolox equivalent antioxidant capacity (TEAC) assay. Optimization conditions were firstly determined using a single-factor experiment, and response surface methodology was then used to evaluate interaction of several experimental parameters. Analysis of the coefficient of determination showed that second-order polynomial models produced a highly satisfactory fitting of the experimental data with regard to TEAC values (*R*^2^ = 0.9829, *p* < 0.0001). The optimal conditions were 39.1% ethanol, and extraction for 35.2 min at 59.4 °C. Under these conditions, the maximum TEAC value was 584.9 ± 6.0 μmol Trolox/g DW, which was higher than those obtained by the conventional extracting method (486.4 ± 12.6 μmol Trolox/g DW) and the Soxhlet extraction method (339.1 ± 16.2 μmol Trolox/g DW). The crude extract obtained could be used either as a food additive or in pharmaceuticals for the prevention and treatment of diseases caused by oxidative stress.

## 1. Introduction

Reactive oxygen species (ROS) could cause a myriad of damages to biological systems, and cause many chronic and degenerative diseases, such as cardiovascular disease, cancer, diabetes mellitus, ageing and neurodegenerative diseases [1,2,3]. The scavenging of ROS is thought to be an effective measure to depress the level of oxidative stress of an organism. Previous studies have demonstrated that intake of fruit and vegetables was inversely associated with the risk of many chronic diseases, such as cancer and cardiovascular diseases [4,5,6,7]. Natural antioxidants in fruit and vegetables are considered to be responsible for these health benefits [8,9]. Antioxidants could serve as potential agents for prevention and treatment of oxidative stress-related diseases. Some cereals, medicinal plants, microalgae and flowers were found to contain high contents of natural antioxidants [10,11,12,13]. Especially, the *Osmanthus fragrans* flower has been demonstrated to exhibit very strong antioxidant activity, to have a neuroprotective effect, and to inhibit melanogenesis [14,15]. In addition, the *Osmanthus fragrans* flower is considered one of the four famous traditional flowers of China and is widely cultivated as an ornamental plant today [16]. The *Osmanthus fragrans* flower is also of economic importance because it is commonly added to tea to increase or improve its taste. Consequently, effective extraction of natural antioxidants from the *Osmanthus fragrans* flower is helpful for its more widely utilization.

Ultrasound-assisted extraction (UAE) was proved to be a useful method for extracting natural antioxidants [17,18]. Recently, UAE has been extensively used on bioactive compounds with promising results. Compared to other traditional extraction techniques, UAE could save extracting time and energy input by increasing extraction efficiency and reducing solvent consumption [19,20]. UAE has been successfully used in biochemical and biotechnological processes related to food systems.

The traditional single-factor experiment assumes that various parameters do not interact, and the process response is thus a direct function of the single varied parameter. Hence, the interaction among various factors has been ignored, and the chance of approaching a true optimum has been impossible [21]. Response surface methodology (RSM) could evaluate effects of several process variables and their interactions on response variables [22]. Thus, RSM is an effective statistical technique, which could be used to explore the optimal conditions for different complex processes [23,24,25]. In this study, an UAE method was developed to extract natural antioxidative ingredients from the *Osmanthus fragrans* flower, and several parameters were optimized using both a single-factor experiment and RSM.

## 2. Results and Discussion

### 2.1. The Results from Single Factor Experiment

The purpose of a single-factor experiment was to evaluate the effect of each factor on antioxidant capacity of the extract from the *Osmanthus fragrans* flower under ultrasound treatment, and to analyze the influence of four different variables.

**Figure 1:**
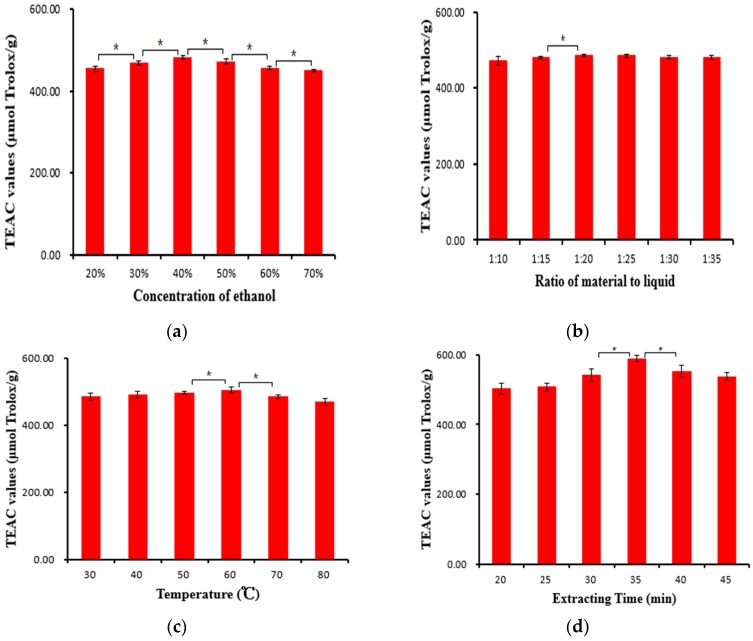
Effects of ethanol concentration (**a**); ratio of material to liquid (**b**); extracting temperature (**c**) and extracting time (**d**) on the extraction efficiencies. Note: * Significant difference (*p* < 0.05).

*Effect of Concentration of Ethanol:* Ethanol is a nontoxic organic solvent for human beings and the environment. Water is an inexpensive solvent and has been widely used in the food industry. Sometimes, the extraction efficiency could be improved using a mixture solvent of ethanol and water [26]. Therefore, ethanol and water were chosen as extracting solvents. The effect of the concentration of ethanol on the extraction efficiencies was tested with a ratio of material to liquid of 1:24 and extracting time of 30 min at 30 °C, and the results are shown in Figure 1a. When the concentration of ethanol increased from 20% to 40%, the extraction efficiencies increased with an increase in ethanol (*p* < 0.05), which was followed by a decrease with an increase in ethanol from 40% to 70% (*p* < 0.05). The results indicated that 40% ethanol was suitable for the extraction of antioxidative ingredients from the *Osmanthus fragrans* flower. Thus, the subsequent experiments were carried out with 40% ethanol.

*Effect of the Ratio of Material to Liquid:* The effect of the ratio of material to liquid on the extraction efficiencies of antioxidative ingredients from the *Osmanthus fragrans* flower was investigated with ethanol concentration of 40% and extracting time of 30 min at 30 °C, and the results are displayed in Figure 1b. When the ratio of material to liquid decreased from 1:10 to 1:20, the extraction efficiencies increased with the decrease of the ratio of material to liquid (*p* < 0.05). When the ratio of material to liquid decreased from 1:20 to 1:35, the extraction efficiencies were almost unchanged (*p* > 0.05). The reason was that a lower ratio of material to solvent could cause a greater concentration difference that accelerated mass transfer and facilitated the diffusion. However, after the mass transfer process reached its maximum, a further decrease of material to solvent ratio could hardly enhance anymore [27,28]. Therefore, the ratio of material to liquid at 1:20 was chosen as the optimal condition for the subsequent experiments.

*Effect of Extracting Temperature:* The effect of extracting temperature on the extraction efficiencies of antioxidative ingredients from the *Osmanthus fragrans* flower was investigated with a ratio of material to liquid of 1:20, ethanol concentration of 40% and extracting time of 30 min, and the results are shown in Figure 1c. The extraction efficiencies significantly increased when the temperature increased from 30 to 60 °C (*p* < 0.05), and the extraction efficiencies then decreased from 60 to 80 °C (*p* < 0.05). The highest extraction efficiency could be obtained at 60 °C. Increasing the extracting temperature could increase diffusivity of the solvent into cells and enhance desorption and solubility of target compounds from the cells, which would result in an increase in extraction efficiencies [29]. Nevertheless, some bioactive compounds from plants could be decomposed at high temperatures, which would lead to the decrease in extraction efficiencies [29,30]. This result indicated that the extracting temperature could influence the recovery of antioxidative ingredients during liquid-solid extraction [31]. Thus, 60 °C was chosen as optimal extraction temperature for the subsequent experiments.

*Effect of Extracting Time:* The effect of extracting time on the extraction efficiencies of antioxidative ingredients from the *Osmanthus fragrans* flower was investigated with a ratio of material to liquid of 1:20, an ethanol concentration of 40% and an extracting temperature of 60 °C, and the results are shown in Figure 1d. There was an increase in the extraction efficiencies from 20 to 35 min (*p* < 0.05), and the extraction efficiencies decreased from 35 to 45 min (*p* < 0.05). The maximum extraction efficiency could be obtained at 35 min. The results indicate that ultrasound might accelerate the establishment of equilibrium for dissolution of the target compounds by disrupting the matrix cell walls to promote the release of bioactive compounds. However, prolonged time induced the degradation of phenolic, and led to the decrease of TEAC values [26]. This could explain the decrease after 35 min.

### 2.2. The Results from Response Surface Methodology

*Experimental Design and Results:* The effects of three independent variables *X*_1_ (concentration of ethanol), *X*_2_ (temperature) and *X*_3_ (extracting time), which have the most influence on extracting efficiency of dependent variable (TEAC values) based on a single-factor experiment, were investigated using central composite design at five levels. The lower, middle and upper levels of the three independent variables employed in the RSM were selected based on values obtained in single-factor experiments (Table 1). Twenty combinations of the independent variables, selected per experimental design for three parameters and the results, are shown in Table 2. Results showed that the TEAC values ranged from 556.8 to 590.8 μmol Trolox/g DW.

**Table 1:** Independent variables and their levels used for central composite design.

Independent Variable	Units	Symbol	Coded Levels				
			−1.68	−1	0	1	1.68
Concentration of ethanol	% (*v*/*v*)	*X*_1_	23.2	30	40	50	56.8
Temperature	°C	*X*_2_	43.2	50	60	70	76.8
Extracting time	min	*X*_3_	26.6	30	35	40	43.4

**Table 2:** The central composite design with experimental values of the investigated responses.

Standard Order ^a^	Run Order ^b^	*X*_1_	*X*_2_	*X*_3_	Response *Y*
		Concentration of Ethanol (%)	Temperature (°C)	Extracting Time (min)	TEAC Values (μmol Trolox/g)
1	1	30.0	50.0	30.0	569.2
2	5	50.0	50.0	30.0	566.7
3	10	30.0	70.0	30.0	556.8
4	13	50.0	70.0	30.0	560.8
5	4	30.0	50.0	40.0	565.2
6	15	50.0	50.0	40.0	562.3
7	9	30.0	70.0	40.0	567.7
8	6	50.0	70.0	40.0	564.2
9	8	23.2	60.0	35.0	568.2
10	14	56.8	60.0	35.0	559.1
11	18	40.0	43.2	35.0	565.2
12	17	40.0	76.8	35.0	564.7
13	2	40.0	60.0	26.6	565.7
14	3	40.0	60.0	43.4	568.2
15	19	40.0	60.0	35.0	587.9
16	16	40.0	60.0	35.0	590.8
17	12	40.0	60.0	35.0	587.9
18	20	40.0	60.0	35.0	586.4
19	7	40.0	60.0	35.0	586.4
20	11	40.0	60.0	35.0	587.9

Note: ^a^ No randomized, ^b^ Randomized.

*Fitting the Model:* Multiple regression equations were generated relating response variables to coded levels of the independent variables. Multiple regression coefficients were determined by employing the least squares technique to predict quadratic polynomial models for antioxidative ingredients from the *Osmanthus fragrans* flower [24]. The data pertaining to coded levels of the independent and response variables were analyzed to get a regression equation as follows:

Y = 587.87 − 1.48X_1_ − 1.07X_2_ + 0.74X_3_ + 0.74X_1_X_2_ − 0.99X_1_X_3_ + 2.83X_2_X_3_ − 8.51X_1_^2^ − 8.04X_2_^2^ − 7.34X_3_^2^

Table 3 presented the ANOVA for response surface quadratic polynomial models. The statistical significance of the regression equation was checked by the *F* test. The models had a very high *f* value (64.0) and a low *p* value (*p* < 0.0001), which indicated that the model was highly significant. The coefficient of determination (*R*^2^) for predicted model was 0.9829, while the adjusted determination coefficient (Adj. *R*^2^) value was 0.9676. The fitness of the model was studied through the lack of fit test. The *F* value of 2.2 and *p* value of 0.2016 indicated the suitability of models to accurately predict the variations. All of these results suggested that the model was adequate for predicting within the range of the variables employed [32].

**Table 3:** ANOVA for response surface models.

Term	Sum of Squares	Degrees of Freedom	Mean Square	*F* Value	*p* Value
Model	2427.7	9	269.7	64.0	<0.0001 ^b^
Concentration of ethanol, *X*_1_	30.1	1	30.1	7.1	0.0235 ^a^
Temperature, *X*_2_	15.7	1	15.7	3.7	0.0828 ^a^
Extracting time, *X*_3_	7.4	1	7.4	1.8	0.2145
*X*_1_*X*_2_	4.4	1	4.4	1.0	0.3326
*X*_1_*X*_3_	7.8	1	7.8	1.8	0.2044
*X*_2_*X*_3_	64.2	1	64.2	15.2	0.0029 ^b^
*X*_1_^2^	1044.7	1	1044.7	247.9	<0.0001 ^b^
*X*_2_^2^	930.5	1	930.5	220.8	<0.0001 ^b^
*X*_3_^2^	776.1	1	776.1	184.1	<0.0001 ^b^
Residual	42.2	10	4.2		
Lack of Fit	29.0	5	5.8	2.2	0.2016
Pure Error	13.1	5	2.6		
Cor Total	2469.9	19			
*R*^2^	0.9829				
Adj. *R*^2^	0.9676				

Note: ^a^ Significant at 10%, ^b^ Significant at 1%.

**Figure 2:**
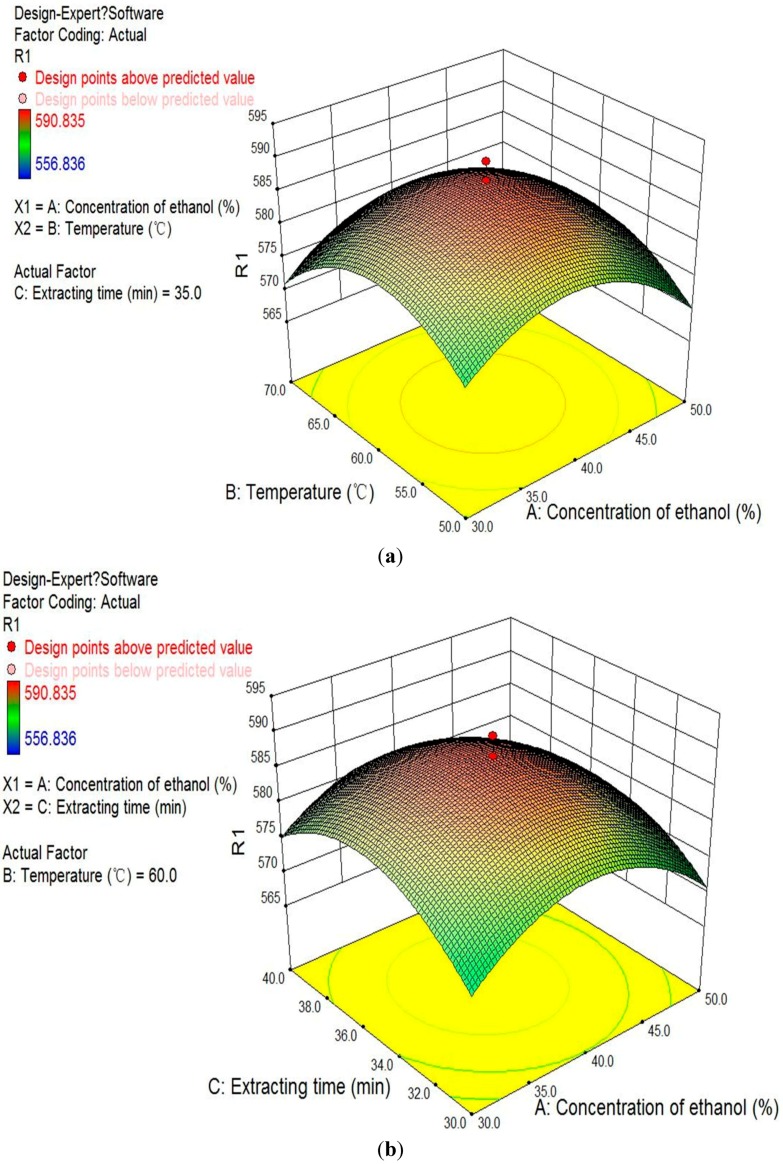

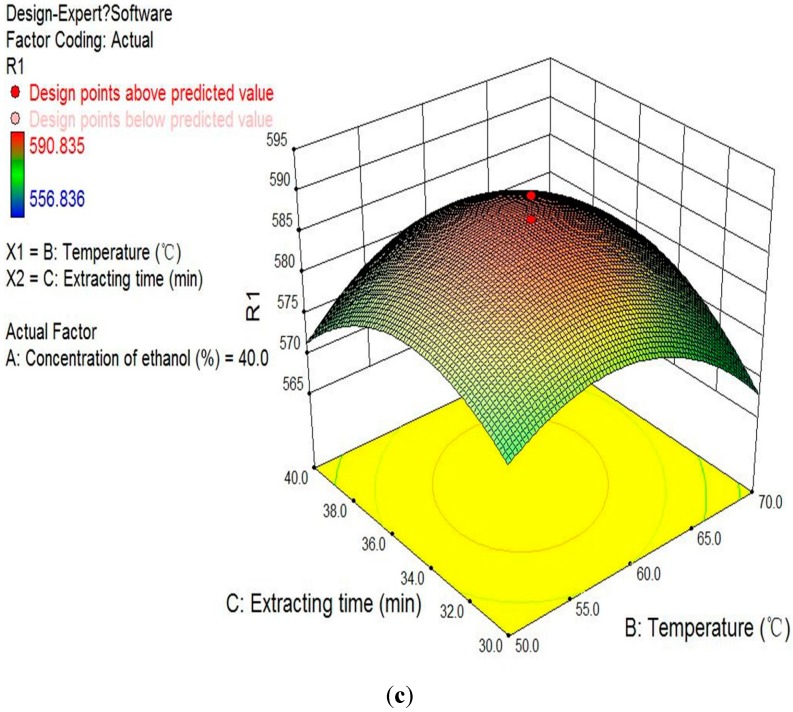
Response surfaces for the combined effects of concentration of ethanol and extracting temperature (**a**); concentration of ethanol and extracting time (**b**); and extracting temperature and extracting time (**c**).

*Analysis of Response Surfaces:* Since the model has shown lack of fit to be insignificant, the responses were sufficiently explained by the regression equation. The regression models allowed the prediction of the effects of the three parameters on antioxidative ingredients from the *Osmanthus fragrans* flower. The relationship between independent and dependent variables was illustrated in three-dimensional representations of the response surfaces. The response surface was generated as a function of concentration of ethanol (23.2%–56.8%) and temperature (26.6–76.8 °C) while keeping the extracting time at 35 min (Figure 2a). The concentration of ethanol demonstrated a positive influence on the response variables. Due to the change of solvent polarity with the change of ethanol concentration, TEAC values increased at a low concentration of ethanol and then decreased with further increase of ethanol concentration [33]. Increased temperature would increase the response variables at first. With further increases in temperature, decreases in the TEAC values were observed. As shown in Figure 2b, the extraction efficiencies significantly increased when the extracting time prolonged before 35 min, and the extraction efficiencies then decreased after 35 min. When the concentration of ethanol was fixed (Figure 2c), both extraction temperature and extracting time showed a strongly positive influence on the response variables.

*Verification Experiments:* It was decided that, in order to obtain the highest TEAC values, the optimum process condition should be investigated. The optimum levels of the independent variables were generated by analyzing the quadratic polynomial regression equations. The optimal extraction condition that provided a maximum TEAC value of 588.0 μmol Trolox/g DW was predicted as follows: 39.1% for concentration of ethanol, 59.4 °C for extracting temperature, and 35.2 min for extracting time. Verification experiments were performed at the predicted conditions, and experimental values were well matched to the predicted value, confirming the validity and adequacy of the predicted models. Moreover, the verification experiments also proved that the predicted values of TEAC by the model could be satisfactorily achieved within a 95% confidence interval of experimental values.

### 2.3. Comparison of Ultrasound-Assisted Extraction with Other Methods

The TEAC values were 486.4 ± 12.6, 339.1 ± 16.2 and 584.9 ± 6.0 μmol Trolox/g for the conventional extracting method, the Soxhlet method and UAE, respectively. The results indicated that UAE was the most efficient extracting method among the three methods. Although the extracting parameters for the conventional extracting method and the Soxhlet method could be not the optimal, these conditions are often used [34,35]. In the literature, Horzic *et al.* [19] had reported that UAE could enhance heat and mass transfer by disrupting the matrix cell walls to promote the release of bioactive compounds. That could explain why UAE had higher extraction efficiency than the conventional extracting method and the Soxhlet extraction method.

## 3. Experimental Section

### 3.1. Chemicals

2,2′-azinobis(3-ethylbenothiazoline-6-sulphonic acid) diammonium salt (ABTS) and 6-hydroxy-2,5,7,8-tetramethylchromane-2-carboxylic acid (Trolox) were purchased from Sigma-Aldrich (St. Louis, MO, USA). Ethanol and potassium persulfate were obtained from Kelong Chemical Factory (Chengdu, China). All chemicals used in the experiments were of analytical grade, and deionized water was used.

### 3.2. Instruments

The ultrasound-assisted extraction was carried out in a KQ-600E ultrasonic device (Changzhou Nuoji Instrument Company, Changzhou, China) with an electric power of 600 W, heating power of 800 W, and frequencies of 40 kHz, equipped with a digital timer and a temperature controller.

### 3.3. Sample Treatment

The *Osmanthus fragrans* flower was purchased from Guangxi, China. The flower was ground into fine particles (96 mesh) using a special grinder for food processing, and then stored at −20 °C in a refrigerator.

An accurate amount (about 0.25 g) of these particles was mixed with an appropriate amount of extraction solvent. The tube with the sample was immersed into the water bath in the ultrasound device, and irradiated for the pre-set extraction temperature and time [36]. After extraction, the sample was centrifuged at 4200 *g* for 30 min, and the supernatant was obtained and stored at −20 °C until used.

### 3.4. Trolox Equivalent Antioxidant Capacity (TEAC) Assay

The TEAC assay was carried out according to the method established in the literature with slight modifications [37]. Briefly, the ABTS^•+^ stock solution was prepared from 7 mmol/L ABTS and 2.45 mmol/L potassium persulfate in a volume ratio of 1:1, and then incubated in the dark for 16 h at room temperature and used within 2 days. The ABTS^•+^ working solution was prepared by diluting the stock solution to an absorbance of 0.70 ± 0.05 at 734 nm. All samples were diluted to provide approximately 20%–80% inhibition of the blank absorbance. One hundred microliters of the diluted sample was mixed with ABTS^•+^ working solution (3.8 mL); after 6 min of incubation at room temperature, the absorbance of the reaction mixture was measured at 734 nm, and the percent of inhibition of absorbance at 734 nm was calculated. Trolox was used as a reference standard, and the results were expressed as µmol Trolox/g dry weight of flower.

### 3.5. Experimental Design

To evaluate the effect of each factor on antioxidant capacity of the extract from the *Osmanthus fragrans* flower under ultrasound treatment, a single-factor experimental design was firstly adopted for analyzing the influence of four different variables. The effects of the concentration of ethanol (20%, 30%, 40%, 50%, 60%, and 70%), material/solvent ratio (1:10, 1:15, 1:20, 1:25, 1:30, and 1:35 g/mL), extraction temperature (30, 40, 50, 60, 70, and 80 °C), and extracting time (20, 25, 30, 35, 40, and 45 min) were investigated separately on the basis of extraction efficiency.

Optimization of the UAE of antioxidative ingredients from the *Osmanthus fragrans* flower was further carried out using RSM [24]. A three-factor and a five-level central composite design (CCD) consisting of twenty experimental runs was employed including six replicates at the center point. Data pertaining to three independent variables and one response variable were analyzed to get a second-order polynomial model as follows:

$$Y=\beta_{0}+\overset{3}{\underset{i=1}{\sum }}\beta_{i}X_{i}+\overset{3}{\underset{i=1}{\sum }}\beta_{\text{ii}}X_{i}^{2}+\sum^{\text{​}}\overset{3}{\underset{i<j=1}{\sum }}\beta_{\text{ij}}X_{i}X_{j}$$

where β_0_, β_i_, β_ii_, and β_ij_ (i ≠ j) are the regression coefficients for intercept, linear, quadratic and interaction terms, respectively, and X_i_, and X_j_ are the independent variables [23].

### 3.6. Other Extracting Methods for Comparison

*Conventional Extracting Method:* Particles of the *Osmanthus fragrans* flower (0.250 g) were mixed with an appropriate amount of extraction solvent, and extracted at 37 °C for 24 h in a shaking water bath. Then, the samples were detected by TEAC assay.

*The Soxhlet Extraction Method:* The particles of the *Osmanthus fragrans* flower (1.500 g) were kept on Whatman filter paper. The solvent was heated at 95 °C in a Soxhlet extractor, and the antioxidant ingredient was extracted into the solvent (150 mL) during percolation. After 4 h of evaporation/condensation/percolation of solvent through particles, the round-bottom flask was taken out, and the solvent was detected by TEAC assay [38].

### 3.7. Statistical Analysis

All the experiments were performed in triplicate, and the mean value was reported. Statistical analysis was performed using SPSS 19.0, Design Expert 8.06 and Excel 2007.

## 4. Conclusions

An UAE method was developed for the extraction of antioxidative ingredients from the *Osmanthus fragrans* flower. The RSM was used for the optimization of UAE of antioxidative ingredients from the *Osmanthus fragrans* flower. The high correlation of the model (*R*^2^ = 0.9829) indicated that it accurately expressed the influence of independent variables on the response measured. The model showed that the highest amount of antioxidative ingredients from the *Osmanthus fragrans* flower could be obtained using 39.1% ethanol as a solvent and extraction for 35.2 min at 59.4 °C. The proposed UAE was more efficient than the conventional extracting method and the Soxhlet extraction method. The crude extract obtained could be used either as a food additive or in pharmaceuticals for the prevention and treatment of diseases caused by oxidative stress.
